# The Georgia Temperance Crusader for 1859

**Published:** 1858-11

**Authors:** 


### THE GEORGIA TEMPERANCE CRUSADER FOR 1859.

We are much pleased to learn by a circular received from the proprietor, that the Temperance Crusader is about to be removed from Penfield to Atlanta. We commend this paper in the most decided manner to the regard and support of our readers, not only on account of its temperance features, but as a literary paper of a high order of merit.

Though it already possesses a large circulation, we hope to see it doubled in a short time after its removal.

The subscription price is $2. All communications to be addressed to John H. Seals, Proprietor, Atlanta, Ga.

